# Synthesis and Application of Silver Nanoparticles for Caries Management: A Review

**DOI:** 10.3390/ph17101264

**Published:** 2024-09-25

**Authors:** Iris Xiaoxue Yin, Veena Wenqing Xu, Grace Yuchan Xu, Ollie Yiru Yu, John Yun Niu, Chun Hung Chu

**Affiliations:** Faculty of Dentistry, University of Hong Kong, Hong Kong, China; irisxyin@hku.hk (I.X.Y.); u3008489@connect.hku.hk (V.W.X.); gracexu1@connect.hku.hk (G.Y.X.); ollieyu@hku.hk (O.Y.Y.); niuyun@hku.hk (J.Y.N.)

**Keywords:** silver nanoparticles, caries, nanoparticles synthesis, antimicrobial properties

## Abstract

Silver nanoparticles have unique physical, chemical, and biological properties that make them attractive for medical applications. They have gained attention in dentistry for their potential use in caries management. This study reviews the different synthesis methods of silver nanoparticles and the application of them for caries management. Silver nanoparticles are tiny silver and are typically less than 100 nanometres in size. They have a high surface area-to-volume ratio, making them highly reactive and allowing them to interact with bacteria and other materials at the molecular level. Silver nanoparticles have low toxicity and biocompatibility. Researchers have employed various methods to synthesise silver nanoparticles, including chemical, physical, and biological methods. By controlling the process, silver nanoparticles have defined sizes, shapes, and surface properties for wide use. Silver nanoparticles exhibit strong antibacterial properties, capable of inhibiting a broad range of bacteria, including antibiotic-resistant strains. They inhibit the growth of cariogenic bacteria, such as *Streptococcus mutans*. They can disrupt bacterial cell membranes, interfere with enzyme activity, and inhibit bacterial replication. Silver nanoparticles can inhibit biofilm formation, reducing the risk of caries development. Additionally, nano silver fluoride prevents dental caries by promoting tooth remineralisation. They can interact with the tooth structure and enhance the deposition of hydroxyapatite, aiding in repairing early-stage carious lesions. Silver nanoparticles can also be incorporated into dental restorative materials such as composite resins and glass ionomer cements. The incorporation can enhance the material’s antibacterial properties, reducing the risk of secondary caries and improving the longevity of the restoration.

## 1. Introduction

Dental caries, also known as tooth decay, is a chronic illness caused by various factors, such as cariogenic microorganisms, fermentable carbohydrates, tooth surfaces, and the passage of time [1]. Enamel is the hardest substance in human tissue. It still can undergo dissolution in acidic oral environments, leading to the destruction of tooth structure and the formation of dental caries.

Dental caries is the most prevalent chronic oral disease and the fourth most expensive to treat worldwide, according to the World Health Organisation [2]. Dental caries is hard in terms of self-limitation. If left untreated, dental caries can advance, causing discomfort, infection, and tooth loss. Fortunately, preventive strategies have been considerably progressed in recent decades. Various dental materials, including metals, ceramics, polymers, and hybrids, are employed for caries management [3,4,5].

Nanotechnology has revealed potential in the development of anti-caries agents through the utilisation of nanoparticles [6]. Nanoparticles are tiny particles with sizes ranging from 1 nm to 100 nm [7]. They possess unique physical properties, such as homogeneity, conductivity, or distinctive optical properties, which make them desirable in the fields of biology and material science. 

The biomedical activities of nanoparticles differ from those of regular size due to their high surface area-to-volume ratios [8]. The development of nanoparticles for medical care is a critical aspect of nanotechnology applications. The use of nanoparticles has had a significant impact on people’s health and daily lives over the past decades.

Among the various types of nanoparticles, silver nanoparticles have exhibited substantial potential in biomedical applications [9]. Silver nanoparticles can enhance medical imaging by providing more detailed visual representations of cellular processes, aiding in medical diagnostics by serving as molecular contrast agents, and improving drug delivery by increasing solubility and bioavailability [10,11,12]. In cancer therapies, silver nanoparticles can eliminate drug-resistant tumour cells and enhance the effectiveness of treatment by targeting and transporting anticancer medications specifically to tumour tissues [13].

Silver nanoparticles are also recognised for their exceptional antimicrobial properties, low toxicity, and cost-effectiveness. They can be used at low doses and do not promote microbial resistance [14]. Various antimicrobial actions have been proposed to explain the reason for the excellent antibacterial effect of silver nanoparticles [15]. Silver nanoparticles release silver ions that penetrate microbial membranes, causing disruption to DNA replication and protein synthesis. Respiratory enzymes may also be activated by silver ions, which can result in cell lysis. Silver nanoparticles can accumulate in the pits of cell walls and denature the cell envelopment. Additionally, they can infiltrate microbial cell walls and cytoplasmic membranes, resulting in the destruction of both the cell wall and cytoplasmic membrane [16]. 

Researchers have investigated using silver nanoparticles to improve the antimicrobial effect of dental materials for clinical use [17]. For caries management, researchers have developed silver nanoparticles using various synthesis strategies. The synthesised silver nanoparticles have shown enormous potential in managing dental caries by preventing biofilm formation and regulating the balance between demineralisation and remineralisation.

Silver nanoparticles enhance dental adhesives and orthodontic brackets for preventing enamel caries, a common orthodontic treatment complication [18]. Incorporating silver nanoparticles into restorative materials can effectively prevent secondary caries, a prevalent factor contributing to restoration failure. Nonetheless, a thorough investigation of silver nanoparticles synthesised for caries management is required. The objective of this study is to present a succinct review of silver nanoparticles synthesised for caries management.

## 2. Synthesis of Silver Nanoparticles

Researchers have employed various methods to synthesise silver nanoparticles, which can be divided into chemical, physical, and biological synthesis methods. The chemical reduction method is the most prevalent method to synthesise silver nanoparticles for caries management. Reducing agents, such as sodium borohydride, ascorbic acid, or citric acid, reduce silver ions to silver nanoparticles. 

Researchers are also exploring physical and biological methods, such as laser ablation and green synthesis using plant extracts or microorganisms, as alternative approaches [19]. Controlling the process allows for low-cost synthesis of homogenous silver nanoparticles with controlled size, morphology, and function for dental applications. Table 1 summarises the advantages and disadvantages of chemical, physical, and biological synthesis methods for developing silver nanoparticles.

### 2.1. Chemical Methods

Chemical methods are one of the most common ways to synthesise silver nanoparticles due to their simple operation [32]. To synthesise silver nanoparticles, chemical methods use water or organic solvents. This procedure typically uses three primary constituents, including silver precursors, reducing agents, and stabilising/capping agents [33]. For the chemical synthesis of silver nanoparticles, silver nitrate commonly serves as a silver precursor due to its desirable characteristics, including its low cost. Various chemical reducing agents can reduce silver ions to silver nanoparticles, such as sodium citrate, ascorbate, sodium borohydride, and elemental hydrogen [34]. The reduction of silver salts essentially consists of two stages: nucleation and subsequent growth. Silver ions receive electrons from reducing agents and are subsequently converted to metallic form. The metallic silver then agglomerates to form silver nanoparticles. Thus, chemical methods are basically a “bottom-up” approach to synthesising silver nanoparticles. Utilising protective agents is crucial for stabilising dispersive nanoparticles during the synthesis process [35]. The stabilising agents absorb onto or bind to the surfaces of nanoparticles, protecting them from aggregation. Surfactants with various functionalities, such as thiols, amines, acids, and alcohols, can cap on the surfaces of silver nanoparticles to enhance their stability and protect them from sedimentation, agglomeration, or losing their defining surface characteristics [36].

Chemical methods have a significant advantage in terms of high yield in contrast to physical methods. Chemical methods require more practical and convenient apparatus compared to physical and biological methods [10]. Nevertheless, the produced nanoparticles may not achieve the anticipated level of purity, as their surfaces were detected to be contaminated with chemicals. Furthermore, the production of silver nanoparticles with precisely determined sizes is challenging, necessitating an additional procedure to avoid particle aggregation. Moreover, the chemical substances employed in the production of silver nanoparticles, including citrate, borohydride, and thio-glycerol, are toxic and hazardous [37,38].

To address the shortcomings of traditional chemical reduction methods, researchers developed alternative methods, such as cryochemical synthesis, electrochemical reduction, sonochemical synthesis, photoinduced reduction, and thermal decomposition methods. Cryochemical synthesis is a method using low-temperature chemical processes to develop nanomaterials [21]. The low-temperature condition minimises the potential for oxidation or other chemical reactions and produces silver nanoparticles with a high degree of purity. Electrochemical reduction is a technique reducing silver ions to silver nanoparticles at the electrode surface by potentiostatic or galvanostatic polarisation [22]. Adjusting electrochemical parameters allows for large-scale production of silver nanoparticles, with control over nanoparticle size and shape.

Sonochemical synthesis uses ultrasound to generate acoustic cavitation in liquid, which leads to high pressure and temperature followed by a high rate of cooling [23]. It can synthesise shape- and size-selective nanoparticles. Photoinduced reduction uses different light sources, such as ultraviolet, white, blue, cyan, green, and orange light, to initiate the reduction reaction of silver ions [24]. This method allows for precise control of the reaction kinetics and results in highly uniform nanoparticles. Furthermore, the photoinduced method has the ability to transform silver nanospheres into triangular silver nanocrystals [39]. Thermal decomposition uses high temperatures to decompose the silver complex to produce silver nanoparticles [25]. It is an efficient technique for the large-scale production of silver nanoparticles.

Researchers have used chemical methods to synthesise silver nanoparticles on graphene oxide. These silver nanoparticle composites are chemically stable and exhibit antibacterial activity against cariogenic bacteria. They inhibit the progression of enamel caries [20]. Chemically synthesised silver nanoparticles can be combined with fluoride to form nano silver fluoride. Studies have reported that nano silver fluoride inhibited cariogenic bacteria and prevented early childhood caries [40,41]. Furthermore, researchers incorporated chemically synthesised silver nanoparticles into resin composites and glass ionomer cement. They used fissure sealant containing silver nanoparticles to inhibit demineralisation and promote remineralisation [42]. Researchers also coated the chemically synthesised silver nanoparticles on orthodontic brackets to prevent enamel caries [43].

### 2.2. Biological Methods

To address the chemical methods’ limitations, biological methods have emerged as viable alternatives. Biological methods, which avoid the involvement of toxic and hazardous chemicals in the synthesis process of silver nanoparticles, have received significant attention in the last decade [44]. The biochemical-mediated synthesis of silver nanoparticles has demonstrated its simplicity, cost-effectiveness, reliability, and environmental friendliness [45]. The synthesis of silver nanoparticles with controllable sizes was achieved by employing biological materials such as bacteria, fungi, plant extracts, and small biomolecules. Studies have reported the biological synthesis of silver nanoparticles using green, cost-effective, and biocompatible methods without the use of toxic chemicals. Bacteria such as *Pseudomonas stutzeri*, *Lactobacillus strains*, and *Bacillus licheniformis* can be used as biomaterials to produce silver nanoparticles [46,47,48]. The formation of silver nanoparticles can occur on the surface of the cytoplasmic cell membrane, inside the cytoplasm, and outside of the cells. It might be because of the bio-reduction of the silver ions by enzymes in the cytoplasm and on the cytoplasmic membrane [45].

It is possible for the silver nuclei that are synthesised outside of the cell wall to enter the cell and combine into larger-sized nanoparticles. Fungi have also been investigated for the synthesis of silver nanoparticles, for example, *Fusarium oxysporum* and *Aspergillus flavus* [49]. The bio-reduction of the silver ions takes place on the surface of the fungi. Proteins play a role in the formation and stabilisation of silver nanoparticles. The plant extracts from green tea, alfalfa, lemongrass, and geranium are abundant in polyphenols and flavonoids. They can be reductants to reduce silver ions, as well as scaffolds to direct the formation of silver nanoparticles [26,27,50]. Biologically synthesised silver nanoparticles using plant extracts may have stronger antibacterial properties than chemically synthesised silver nanoparticles using reductants such as sodium borohydride [51]. Plant extracts exhibit antibacterial properties against cariogenic bacteria. They can synergistically inhibit bacteria with silver nanoparticles. Additionally, plant extracts can stabilise and prevent the aggregation of silver nanoparticles, maintaining the antibacterial properties [52].

Researchers have also used biopolymers, proteins/enzymes, amino acids, and polysaccharides to synthesise silver nanoparticles [53]. These biomaterials allow researchers to control the shape, size, and homogeneity of nanoparticles. The advantages of biological methods include a wide variety of biological resources, reduced time required, high density, stability, and the promising solubility of synthesised nanoparticles in aqueous solutions. Importantly, they are ecologically and financially sustainable with minimal toxicity.

Researchers have synthesised silver nanoparticles using tea extract as a bio-reducing agent, which can impede biofilm formation on dentine surfaces [26]. They have also synthesised nano silver fluoride using glucose. The nano silver fluoride can promote dentine remineralisation [54]. Furthermore, researchers have synthesised silver nanoparticles on an orthodontic elastomeric module using plant extract to prevent enamel caries. This orthodontic elastomeric module can inhibit cariogenic bacteria without compromising physical properties [55].

### 2.3. Physical Methods

Physical methods for synthesising silver nanoparticles include laser ablation, evaporation-condensation, electrical irradiation, gamma irradiation, and lithography. Laser ablation and evaporation-condensation are the commonly used physical methods for nanoparticle synthesis. Without chemical reagents in solution, laser ablation of bulk silver can produce silver nanoparticles [19]. This approach enables the preparation of pure and uncontaminated metal colloids for subsequent applications. The evapouration-condensation method involves the evapouration of a silver-containing solution followed by the condensation of the vapour to form silver nanoparticles [28]. This method can produce high-purity silver nanoparticles because there are no reducing or stabilising agents during the synthesis process. Although laser ablation and evapouration-condensation have the benefit of not requiring hazardous reducing or stabilising agents, both methods have drawbacks such as low yield, high energy consumption, and a lack of uniform distribution. Electrical irradiation uses high-energy electrical discharges to produce plasma, which then interacts with a silver-containing solution to produce silver nanoparticles [29]. This method can yield silver nanoparticles efficiently, but it requires the use of toxic gases. Gamma irradiation uses high-energy gamma rays to reduce silver ions to form nanoparticles [30]. The lithography method uses lithographic techniques to selectively deposit silver onto specifically patterned areas [31].

Physical methods have been less frequently used to synthesise silver nanoparticles for caries management. Researchers have used the evapouration-condensation method to synthesise silver nanoparticle-loaded amorphous calcium phosphate. They incorporated nanocomposite into dental adhesive to prevent secondary caries [56].

## 3. Silver Nanoparticles Application for Caries Management

Silver nanoparticles have the potential to manage dental caries. Researchers have utilised silver nanoparticles in toothpaste to prevent caries [57]. The combined use of silver nanoparticles and fluoride can remineralise and halt enamel and dentine caries [58]. Silver nanoparticles have been incorporated into restorative materials, including filling resins and adhesives, to inhibit secondary caries [59,60]. The addition of silver nanoparticles into glass ionomer cements can prevent dental caries [61]. According to a clinical trial, a dental sealant containing silver nanoparticles was more effective than a conventional sealant in preventing enamel caries in the first permanent molars [42]. In addition, orthodontic brackets were coated with silver nanoparticles to guard against enamel caries [62]. Researchers have also incorporated silver nanoparticles into the resin of orthodontic materials, including adhesives, elastomeric ligatures, and removable retainers, to prevent caries. Glass ionomers with silver nanoparticles can prevent enamel caries as orthodontic cements.

**Figure 1:**
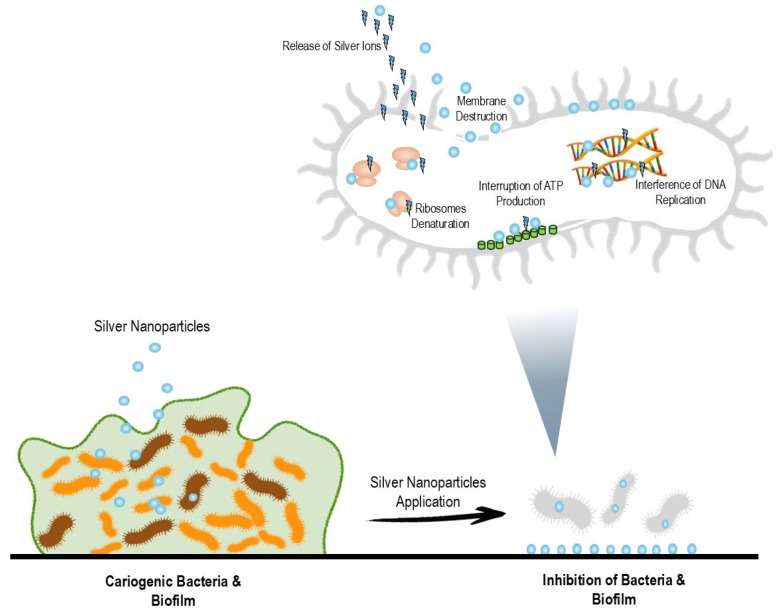
The inhibitory effects of silver nanoparticles on bacterial growth and biofilm formation.

Researchers have applied silver nanoparticles to teeth to prevent dental caries because silver nanoparticles inhibit bacterial growth and biofilm formation (Figure 1). Moreover, silver nanoparticles can infiltrate and lodge into dentinal tubules without aggregation on the dentine surface [63]. They can remain in the dentine structure as a stable and continuous source of silver ions to prevent bacterial invasion. The depth of silver nanoparticles’ infiltration increases with longer exposure times and smaller nanoparticle size. This mechanism explains the long-term anti-caries effectiveness of silver nanoparticles reported in clinical trials [40].

Glass ionomer cement is a commonly used restorative material that releases fluoride for remineralisation. Nevertheless, cariogenic bacteria can invade when microleakage occurs between the tooth and glass ionomer cement interface [64]. The fluoride released from glass ionomer cement is insufficient to suppress bacterial growth. Therefore, researchers have incorporated silver nanoparticles into glass ionomer cement to reduce biofilm formation and prevent secondary caries. Additionally, glass ionomer cement exhibits inferior wear resistance because of voids in the cement matrix. These voids can act as stress raisers and eventually weaken the mechanical properties of the glass ionomer cement. The incorporation of silver nanoparticles into glass ionomer cement can reduce voids. Silver nanoparticles improve the packing of particles within the cement matrix, leading to a more homogeneous and denser structure. Moreover, silver nanoparticles can occupy the empty spaces between the large glass particles and provide an additional bonding site. Researchers have found that silver nanoparticle-containing glass ionomer cement has improved flexural and compressive strengths [65]. The concentration of silver nanoparticles in glass ionomer cement can affect the interaction of glass ionomer cement with the tooth structure. Several studies have shown that using less than 0.5% silver nanoparticles in glass ionomer cement improves its mechanical properties. When the concentration exceeds 0.5%, silver nanoparticles can affect the bond strength of glass ionomer cement with the tooth [65].

Resin composite is another commonly used restorative material with favourable aesthetic and adhesive properties. However, the polymerisation shrinkage of resin composite adversely affects the longevity of restoration. It causes interfacial defects and the marginal microleakage of resin composite [66]. Resin composite lacks antibacterial properties. Thus, biofilm can form on resin composite resulting in secondary caries. The incorporation of silver nanoparticles into resin composite provides antibacterial properties without compromising the mechanical properties. Silver nanoparticles can be well dispersed in the bond interfaces of resin composite without agglomeration. Moreover, silver nanoparticles less than 20 nm in size could infiltrate and occlude dentinal tubules [67]. Silver nanoparticles can also prevent the destruction of bond strength by reducing acid produced by bacteria.

### 3.1. Effect of Silver Nanoparticles on Cariogenic Bacteria

Silver nanoparticles have antibacterial properties against cariogenic bacteria, including *Streptococcus*, *Lactobacillus*, and *Pseudomonas*. *Streptococcus mutans* is the most common cariogenic bacterium found in carious lesions [68]. It is associated with the initiation and development of caries. This bacterium is the most used bacteria for examining the antibacterial efficacy of silver nanoparticles in caries therapy. Silver nanoparticles kill cariogenic bacteria in all studies. However, the findings regarding the minimum inhibitory concentration of silver nanoparticles against cariogenic bacteria varied in different studies [6].

Bacteria gather inside the extracellular matrix to form biofilm. Biofilms can augment the resistance of microbes to antimicrobial drugs by obstructing the transit of drugs. Consequently, the inhibitory impact of biofilm adherence is essential for assessing the antibacterial properties of silver nanoparticles. Single-species biofilm is commonly employed to examine the inhibition properties of silver nanoparticles because of its stability and operational simplicity [26]. Nonetheless, single-species biofilm significantly differs from dental biofilm in vivo. The dental biofilm is a complex biological system consisting of more than 1000 bacterial species [69]. Therefore, some researchers have suggested using the extraction of various microorganisms and the extracellular matrix from patients to culture the microcosm. It can convincingly replicate the intricacy and diversity of the biological biofilm found in the human oral cavity. Therefore, researchers have frequently selected the microcosm model to investigate the antibacterial impact of silver nanoparticles [70].

Researchers have also demonstrated the antimicrobial effect of silver nanoparticles against oral microcosms. Silver nanoparticles can decrease the metabolic activity, lactic acid production, and glucosyltransferase gene expression of biofilm [26,71]. While the microcosm model is unable to completely replicate the real-life oral environment, an in-situ model remains essential for assessing the effectiveness of anti-biofilm measures. Thus, some studies use animal tests or clinical trials to determine the authentic antimicrobial efficiency of silver nanoparticles.

Different antibacterial actions have been suggested, even though the exact mechanism of silver nanoparticles’ antibacterial effects has not been fully revealed (Figure 1). Silver nanoparticles can continuously release silver ions. Silver ions can increase the permeability of the cell membrane and destroy the bacteria envelopment [72]. After silver ions enter the cell membrane, they can deactivate respiratory enzymes and inhibit adenosine triphosphate production. Furthermore, silver ions suppress deoxyribonucleic acid replication and then impact protein synthesis. Silver nanoparticles can eradicate bacteria on their own in addition to releasing silver ions. Silver nanoparticles can anchor and accumulate in the pits of the cell wall [73]. Additionally, the cell membrane becomes denatured when silver nanoparticles accumulate on it. Silver nanoparticles penetrate bacterial cell walls and cause structural damage in the cell membrane due to the nanoscale size of silver nanoparticles.

The antibacterial properties of silver nanoparticles against cariogenic bacteria are dependent on their specific properties. The silver nanoparticles of smaller sizes have a stronger antibacterial effect against cariogenic bacteria [74]. Due to their higher surface-to-volume ratios, smaller silver nanoparticles have a higher propensity to release silver ions. The increased release of silver ions from silver nanoparticles promotes their inhibition effect against the bacteria. Moreover, silver nanoparticles can penetrate dentinal tubules and adhere to their walls. The smaller the silver nanoparticles, the deeper their penetration. The penetration of silver nanoparticles into dentinal tubules may explain their long-term antibacterial effect on dentine surfaces. Furthermore, capping agents have an impact on the anti-cariogenic bacterial activity of silver nanoparticles [75]. The surface modification of silver nanoparticles by capping agents alters the dissolving efficiency of the nanoparticles. The positively charged silver nanoparticles exhibit a significant level of bacterial activity against *Streptococcus mutans* [76]. The cellular membranes of cariogenic bacteria such as *Streptococcus mutans* are negatively charged. Cariogenic bacteria attract positively charged silver nanoparticles due to their mutual electrostatic attraction. Silver nanoparticles effectively suppress both gram-positive and gram-negative bacteria. Generally, gram-positive bacteria are more resistant to silver nanoparticles than gram-negative bacteria [77,78]. Gram-negative bacteria have an outer cell membrane. It serves as a barrier to inhibit the entry of antibiotics into the cells. However, silver nanoparticles can pass through this barrier because of their nanoscale. Silver nanoparticles can disrupt the outer membrane and increase permeability. Furthermore, gram-positive bacteria have a thicker peptidoglycan layer in their cell wall to reduce damage induced by silver nanoparticles.

The formation of biofilm is rapid in the oral environment. The biofilm can significantly hinder the movement of silver nanoparticles. Thus, biofilm can protect bacteria from impact. Research indicates that bacterial viability can be preserved in biofilm after the treatment of silver nanoparticles. Although, at the same concentration, silver nanoparticles can eliminate all planktonic bacteria [17]. Because of its complicated architecture, the biofilm is tolerant of silver nanoparticles. Silver nanoparticles of different sizes, shapes, and synthesis methods have diverse diffusion coefficients. The mobility of silver nanoparticles in biofilm is closely related to their diffusion coefficients [79]. The diffusion coefficient is negatively correlated with the particle’s size. Silver nanoparticles of large sizes find it harder to penetrate biofilm than those of small sizes. Transport through biofilm can be significantly hindered for particles larger than 50 nm. The surface charge and modification of silver nanoparticles can influence their diffusion in biofilm by causing absorption and aggregation. The biofilm can electrostatically interact with charged silver nanoparticles and hinder their penetration of biofilm [80]. Meanwhile, the increased concentration of silver nanoparticles in materials improved their antibacterial properties.

### 3.2. Effects of Silver Nanoparticles on Enamel and Dentine

Silver nanoparticles can inhibit tooth demineralisation under acidic or cariogenic conditions. Researchers have found that silver nanoparticles can make the carious lesion on sound enamel shallower during biofilm challenging. In addition, enamel with artificial caries treated with silver nanoparticles increased in terms of microhardness. Thus, the topical use of silver nanoparticles reduced mineral loss and prevented dental caries on both sound and demineralised enamel [81]. This inhibition effect on demineralisation can be attributed to the suppression of acid production in biofilm by silver nanoparticles. In dentine caries, collagen exposure occurs after the dissolution of mineral content. Silver nanoparticles can protect exposed collagen [82]. The exposed collagen is subject to degradation by bacterial collagenases, as well as proteinases present in saliva and the dentine matrix. Silver nanoparticles can deactivate these enzymes for the conservation of dentine collagen. Then, the conserved collagen can promote remineralisation by acting as a framework for mineral crystal formation. A study using a chemical model found that silver nanoparticles increased the microhardness of enamel caries [83]. Silver nanoparticles have the capability to infiltrate carious lesions and adhere to hydroxyapatite crystals. Moreover, the released silver ions from silver nanoparticles can produce insoluble silver chloride on dental hard tissue [58]. The insoluble silver chloride and precipitated silver nanoparticles raise the mineral density of enamel and dentine.

Even though silver nanoparticles have a positive effect on dental hard tissue, fluoride has a more promising remineralising effect than silver nanoparticles do. Fluoride has been widely used for caries management in practice. Fluoride can react with calcium ions and hydrogen phosphate ions to produce fluorapatite and fluorhydroxyapatite. These chemicals exhibit greater resistance to acidity compared to hydroxyapatite. Therefore, the combination use of silver nanoparticles and fluoride (nano silver fluoride) can enhance the remineralising effect of tooth structure [58]. The microhardness of sound enamel treated with nano silver fluoride is comparable to that of sodium fluoride-treated enamel. Nano silver fluoride also exhibited efficacy on enamel with carious lesions. Nano silver fluoride exhibited a stronger effect on arresting enamel caries than sodium fluoride in the microhardness test. However, optic coherence tomography is unable to distinguish the variation in mineral composition between decayed enamel treated with nano silver fluoride and sodium fluoride. A clinical investigation showed that nano silver fluoride is as efficient at preventing dental cavities in children as silver diamine fluoride [84]. While nano silver fluoride did not show a significant staining effect on a carious lesion, silver diamine fluoride dyed it black. Moreover, the use of silver nanoparticles in restorative materials prevented demineralisation. A resin with silver nanoparticles increased the microhardness of dentine caries after acid challenge [85]. In addition, a clinical trial demonstrated that dental sealant containing silver nanoparticles effectively reduced mineral loss in children’s molars. Nevertheless, some studies did not specify the precise amounts of silver nanoparticles used in their investigations. Therefore, further investigation is required to examine the optimal concentrations of silver nanoparticles for caries prevention.

## 4. Potential Risk

Despite the potential benefits of using silver nanoparticles to prevent dental caries, some risks and concerns need to be addressed. High concentrations of silver nanoparticles can lead to inflammation, oxidative stress, and cell damage [86]. Researchers have explored the nontoxic and low-toxic range of silver nanoparticles’ concentrations to safely use. The size, surface charge, and functionalisation of silver nanoparticles can influence their toxicity. It is crucial to optimise the silver nanoparticles’ size and surface modification to ensure safety and biocompatibility. To minimise toxicity, researchers have developed silver nanoparticles using capping agents. Capped silver nanoparticles displayed lower toxicity than uncapped silver nanoparticles when used on human cells. The widespread use of silver nanoparticles may also raise concerns about their potential environmental impact. Silver nanoparticles can enter the environment through wastewater systems, leading to the accumulation of silver in water bodies, soil, and organisms [86]. The long-term effects of this accumulation on ecosystems and human health are still unknown. To minimise their environmental impact, it is essential to develop strategies for the safe disposal and recycling of dental materials containing silver nanoparticles.

## 5. Conclusions

Silver nanoparticles inhibit bacteria growth and biofilm formation and hence impede the demineralisation of enamel and dentine. Researchers have synthesised silver nanoparticles using chemical, physical, and biological methods for caries management. They incorporated silver nanoparticles into topical fluoride agents and restorative dental materials for caries prevention.

## Figures and Tables

**Table 1:** Chemical, biological, and physical synthesis methods for silver nanoparticles for caries management.

Methods (Ref.)	Reaction Conditions	Advantages	Disadvantages
**Chemical Methods** (*Common methods to synthesise silver nanoparticles)*			
Chemical reduction [20]	Silver precursors Reducing agents Stabilising agents	High yield Simple and low-cost method	Toxic chemicals Low purity Toxic byproducts
Cryochemical synthesis [21]	Silver precursors Reducing agents Stabilising agents Low temperature	Narrow size distribution Little particle agglomeration	Specialised equipment Time-consuming process Low yield
Electrochemical reduction [22]	Silver precursors Reducing agents Stabilising agents Electrodes	Precise control over particle size Precise control over particle shape High yield Simple and low-cost method	Toxic chemicals Wide size distribution Low purity
Sonochemical synthesis [23]	Silver precursors Reducing agents Stabilising agents Ultrasound irradiation	Narrow size distribution High yield	Specialised equipment High energy consumption Risk of agglomeration
Photoinduced reduction [24]	Silver precursors Reducing agents Stabilising agents Light irradiation	Simple and low-cost method Precise control over particle size Precise control over particle shape High purity of nanoparticles	Toxic chemicals Low yield
Thermal decomposition [25]	Silver complex Reducing agents Stabilising agents High temperature Inert gas atmosphere	Simple and low-cost method High purity of nanoparticles Narrow size distribution High yield	High energy consumption Toxic byproducts Limited size control
**Biological Method** (*alternative method to synthesise silver nanoparticles*)			
Bio-reduction [26,27]	Silver precursors Bio-reducing agents Bio-stabilising agents	Environmentally friendly Sustainability Biocompatible and non-toxic.	Low yield Limited size control
**Physical Methods** (*Less frequently used methods to synthesise silver nanoparticles*)			
Laser ablation [19]	Silver bulk Laser irradiation	High purity of nanoparticles Narrow size distribution No chemical reducing agents	High energy consumption Specialised equipment Low yield
Evaporation-condensation [28]	Silver precursors Organic solvents High temperature Vacuum conditions	Low-cost method High yield	Wide size distribution Low purity
Electrical irradiation [29]	Silver precursors Electrical current	High yield Narrow size distribution No chemicals reducing agents	High energy consumption Specialised equipment Potential toxic byproduct
Gamma irradiation [30]	Silver precursors Gamma irradiation	High yield Narrow size distribution No chemicals reducing agents	High energy consumption Specialised equipment Risk of radiation hazards
Lithography [31]	Silver precursors Photoresist Developer Photolithography Etching UV irradiation	Precise control of particle size Precise control of particle shape High purity of nanoparticles Narrow size distribution	Specialised equipment Time-consuming process Low yield

## Data Availability

Not applicable.
